# Partnering with general practitioners to optimize survivorship for patients with lymphoma: a phase II randomized controlled trial (the GOSPEL I trial)

**DOI:** 10.1186/s13063-020-04945-4

**Published:** 2021-01-06

**Authors:** Raymond Javan Chan, Stephanie Buhagiar, Laisa Teleni, Camilla Simonsen, Jane Turner, Courtney Rawson, Nicolas H. Hart, Lee Jones, Louisa Gordon, Ria Joseph, Oluwaseyifunmi Andi Agbejule, Fiona Henderson, Joel Rhee, Marissa Ryan, Christine Carrington, Sally Mapp

**Affiliations:** 1 Princess Alexandra Hospital and Queensland University of Technology (QUT), Brisbane, Queensland Australia; 2 Division of Cancer Services, Princess Alexandra Hospital, Brisbane, Queensland Australia; 3 Division of Cancer Services, Princess Alexandra Hospital and Queensland University of Technology (QUT), Brisbane, Queensland Australia; 4 Faculty of Medicine, University of Queensland and Royal Brisbane and Women’s Hospital, Brisbane, Queensland Australia; 6 Cancer and Palliative Care Outcomes Centre, Queensland University of Technology (QUT), Brisbane, Queensland Australia; 7 QIMR Berghofer Medical Research Institute, Brisbane, Queensland Australia; 8 Cancer and Palliative Care Outcomes Centre, Queensland University of Technology (QUT), Brisbane, QLD Australia; 9 University of Wollongong, Wollongong, Australia

**Keywords:** Lymphoma, Hematology, Shared care, Quality of life, Protocol, Randomized controlled trial

## Abstract

**Background:**

Survival rates for lymphoma are highest amongst hematological malignancies. In 2019, it was estimated that over 6400 Australians were diagnosed with lymphoma, a group of hematological malignancies with a high 5-year survival rate of ~ 76%. There is an increased focus on the promotion of wellness in survivorship and active approaches to reducing morbidity related to treatment; however, current models of follow-up care heavily rely on hospital-based specialist-led care.

Maximizing the potential of general practitioners (GPs) in the ongoing management of cancer is consistent with the national health reform principles and the Cancer Council Australia’s Optimal Care Pathways. GPs are well positioned to provide guideline-based follow-up care and are more likely to address comorbidities and psychosocial issues and promote healthy lifestyle behaviors. This study aims to test the feasibility of the GOSPEL I intervention for implementing an integrated, shared care model in which cancer center specialists and community-based GPs collaborate to provide survivorship care for patients with lymphoma.

**Methods:**

We describe a protocol for a phase II, randomized controlled trial with two parallel arms and a 1:1 allocation. Sixty patients with Hodgkin’s and non-Hodgkin’s lymphoma will be randomized to usual specialist-led follow-up care (as determined by the treating hematologists) or a shared follow-up care intervention (i.e., GOSPEL I). GOSPEL I is a nurse-enabled, pre-specified shared care pathway with follow-up responsibilities shared between cancer center specialists (i.e., hematologists and specialist cancer nurses) and GPs. Outcome measures assess feasibility as well as a range of patient-reported outcomes including health-related quality of life as measured by the Functional Assessment of Cancer Therapy—Lymphoma, patient experience of care, symptom distress, comorbidity burden, dietary intake, physical activity behaviors, financial distress/interference, and satisfaction of care. Safety indicators including hospital admission and unscheduled lymphoma clinic visits as well as process outcomes such as intervention fidelity and economic indicators will be analyzed.

**Discussion:**

This trial is designed to explore the feasibility and acceptability of a new model of shared care for lymphoma survivors. Patient-reported outcomes as well as potential barriers to implementation will be analyzed to inform a larger definitive clinical trial testing the effects and implementation of a shared care model on health-related quality of life of lymphoma survivors.

**Trial registration:**

Australia and New Zealand Clinical Trials Registry ACTRN12620000594921. Registered on 22 May 2020.

## Administrative information

The order of the items has been modified to group similar items (see http://www.equator-network.org/reporting-guidelines/spirit-2013-statement-defining-standard-protocol-items-for-clinical-trials/).

**Table:** 

Title {1}	Partnering with General practitioners to optimize Survivorship for patients with Lymphoma: A phase II randomized controlled trial (The GOSPEL I Trial)
Trial registration {2a and 2b}.	Registry Name: Australia and New Zealand Clinical Trials Registry. Trial Identifier: ACTRN12620000594921. All SPIRIT items can be found within the body of the protocol.
Protocol version {3}	03/08/2020, Version 1.5
Funding {4}	This study is funded by a Health Innovation, Investment and Research Office (HIIRO). Queensland Advancing Clinical Research Fellowship
Author details {5a}	Raymond Javan Chan, Division of Cancer Services, Princess Alexandra Hospital, Metro South Health & School of Nursing, Queensland University of Technology (QUT), Brisbane, Queensland, Australia. Email: raymond.chan@qut.edu.au Stephanie Buhagiar, Lymphoma Clinical Nurse Consultant, Division of Cancer Services, Princess Alexandra Hospital. Metro South Health. Email: stephanie.buhagiar@health.qld.gov.au Laisa Teleni, Research Coordinator, School of Nursing, Queensland University of Technology (QUT), Brisbane, Queensland, Australia. Email: laisa.teleni@qut.edu.au Camilla Simonsen, Division of Cancer Services, Princess Alexandra Hospital, Metro South Health, Brisbane, Queensland, Australia; Senior Research Assistant, Queensland University of Technology (QUT). Email: Camilla.Simonsen@health.qld.gov.au Jane Turner, Faculty of Medicine, University of Queensland & Royal Brisbane and Women’s Hospital, Brisbane, Queensland, Australia. Email: jane.turner@uq.edu.au Courtney Rawson, Division of Cancer Services, Princess Alexandra Hospital, Metro South Health, Brisbane, Queensland, Australia; Senior Research Assistant, Queensland University of Technology (QUT). Email: courtney.rawson@health.qld.gov.au Nicolas H. Hart, Division of Cancer Services, Princess Alexandra Hospital, Metro South Health & School of Nursing, Queensland University of Technology (QUT), Brisbane, Queensland, Australia. Email: nicolas.hart@qut.edu.au Lee Jones, Biostatistician, Queensland University of Technology (QUT). Email: Lee.Jones@qut.edu.au Louisa Gordon, Team Health (Health Economics), QIMR Berghofer Medical Research Institute. Email: louisa.gordon@qimrberghofer.edu.au Ria Joseph, Research Assistant/PhD Candidate, School of Nursing, Queensland University of Technology (QUT), Brisbane, Queensland, Australia. Email: Ria.Joseph@qut.edu.au Oluwaseyifunmi Andi Agbejule, Research Assistant/PhD Candidate, School of Nursing, Queensland University of Technology (QUT), Brisbane, Queensland, Australia. Email: oa.agbejule@qut.edu.au Fiona Henderson, Clinical Nurse Consultant, Division of Cancer Services, Princess Alexandra Hospital, Metro South Health, Brisbane, Queensland, Australia. Email: Fiona.Henderson@health.qld.gov.au Joel Rhee, General Practitioner & Associate Professor of General Practice, University of Wollongong, Wollongong, Australia.. Email: jrhee@uow.edu.au Marissa Ryan, Oncology Pharmacy Team Leader, Cancer Services, Princess Alexandra Hospital , Metro South Health Email: Marissa.ryan@health.qld.gov.au Christine Carrington, Senior Consultant Pharmacist, Cancer Services, Princess Alexandra Hospital, Metro South Health Email: christine.carrington@health.qld.gov.au Sally Mapp, Senior Haematologist and Head of Lymphoma, Division of Cancer Services, Princess Alexandra Hospital, Metro South Health Email: sally.mapp@health.qld.gov.au
Name and contact information for the trial sponsor {5b}	Professor Raymond Chan Division of Cancer Services, Princess Alexandra Hospital, Metro South Health, Brisbane, Queensland, Australia. And School of Nursing and Cancer and Palliative Care Outcomes Centre, Queensland University of Technology, Brisbane, Queensland, Australia. Email: Raymond.chan@qut.edu.au
Role of sponsor {5c}	The sponsor has absolute authority over securing funding, study design, data analysis, interpretation of data, writing of the report, decision to submit the report for publication.

## Introduction

### Background and rationale {6a}

In 2019, it was estimated that over 6400 Australians were diagnosed with Hodgkin’s lymphoma (HL) and non-Hodgkin’s lymphoma (NHL) [1]. Lymphomas are hematological malignancies with high overall 5-year survival rates (~ 76%) resulting in a large cohort of people post therapy or undergoing regular surveillance for incurable but indolent disease not yet warranting treatment. Lymphoma survivors require ongoing survivorship care including monitoring for cancer progression or recurrence, surveillance for secondary/new primary cancer, and management of a range of long-term bio-psycho-social effects from their cancer diagnosis and treatment. Moreover, many cancer survivors are also in need for management of comorbidities [2]. Compared to people without cancer, people with cancer are more likely to develop mental and behavioral problems (2.5 times), circulatory conditions (1.3 times), musculoskeletal conditions (1.4 times), and endocrine system disorders (1.2 times) [2]. These health concerns highlight the importance of a well-integrated, patient-centered model of care that addresses cancer-related survivorship needs as well as comorbidities for people who have been diagnosed with lymphoma.

The current models of cancer care in Australia are mostly specialist-led and focus on surveillance for disease recurrence, rather than holistic care needs. These models of care limit integration between specialist institutions and the patient’s community-based general practitioner (GP), also called family physician or primary care physician in some countries. With the ever-growing and aging population of cancer survivors, specialist-led follow-up is not optimal as it does not encourage an integrated, patient-centered approach in which comorbidities and long-term effects experienced by cancer survivors can be effectively managed. Specialist-led follow-up does not capitalize on the expertise of GPs, and it requires patients living in the rural/regional areas to travel long distances to the cancer centers.

Ongoing care of patients with chronic illnesses such as cancer is core business for general practice. Maximizing the potential of GPs in the ongoing management of cancer is consistent with the national health reform principles [3–5] and the Optimal Care Pathways [6, 7]. In the breast cancer setting, it is clear that GPs can provide guideline-based follow-up care and are more likely to address comorbidities and psychosocial issues and promote healthy lifestyle behaviors compared to cancer specialists [8]. Shared care arrangements might also facilitate a smoother transition to “mainstream” clinical care for patients who have successfully undergone treatment and had several years of follow-up without recurrence. A shared care approach is likely to be more cost-effective than a specialist-led model due to the added benefits from harnessing the expertise of both the cancer specialist as well as the GP. Such an approach, if effective, may further relieve pressures in health services to meet the ever-increasing demand. The recent COVID-19 pandemic has further highlighted the need to explore, implement, and evaluate alternative models of care, enabling a reduction in acute hospital setting presentations and enhanced community GP involvement [9].

Although implementing an integrated, shared care follow-up model is the next logical step for cancer patients with high survival rates, the evidence base supporting this model is less established for lymphoma compared to breast cancer. There are two key reasons that a shared follow-up care model between specialists and GPs is not routinely implemented for lymphoma survivors across Australia. Firstly, randomized controlled trials (RCT) evidence for the effectiveness and cost-effectiveness of a shared care model between specialists and GPs for lymphoma are *not* yet established [10]. Secondly, a number of barriers to shared care between the acute cancer care setting and GPs have been reported [11, 12]. These include, but are not limited to, the lack of a coordinator who drives a shared model involving multiple providers, lack of patient and provider knowledge about the benefits of shared care and how to implement it, insufficient or delayed communication between cancer specialists and GPs, and lack of awareness of available support such as funding models, tools and resources [11–13]. These barriers might be overcome if a specialist cancer nurse (SCN) advises stakeholders of the benefits of shared care (patient and GPs) and facilitates effective and timely care coordination and communication by acting as the conduit between the specialist cancer multidisciplinary team and the GPs at key transition time points (such as completion of treatment) [14].

### Objectives {7}

The objective of the study is to test the feasibility of a prospective, pragmatic, randomized controlled trial (RCT) of the *GOSPEL I* intervention—an integrated, shared care model involving hematologists and GPs for lymphoma follow-up.

### Trial design {8}

This single-center, phase II pilot RCT aims to assess the feasibility of a larger definitive clinical trial. Outcome data will be collected at three points: (*t*_*1*_) baseline (at enrolment), (*t*_*2*_) 6 months, and (*t*_*3*_) 12 months.

## Methods: participants, interventions, and outcomes

### Study setting {9}

This study is to be conducted in Princess Alexandra Hospital—a large, Australian metropolitan tertiary teaching hospital and involves general practices of the surrounding areas.

### Eligibility criteria {10}

Due to the varying follow-up requirements for patients with lymphoma, the study population consists of two groups of patients who require two distinct follow-up pathways. Group one (post-treatment follow-up) consists of patients with a histopathologically confirmed diagnosis of aggressive or indolent lymphoma in the acute post-treatment phase (i.e., within 3 months of completion of chemotherapy). Group two (observation follow-up) consists of patients with a histopathologically confirmed diagnosis of indolent lymphoma followed up in the surveillance clinic who are at least 2 years post-treatment or treatment naïve. In addition to meeting one of the group descriptions above, participants must also meet all of the following criteria to be eligible for inclusion: ≥ 18 years of age, have an Easter Cooperative Oncology Group (ECOG) performance status < 2, be an ambulatory outpatient at the time of recruitment, be able to nominate a GP or GP clinic to be involved in their follow-up, have access to a telephone, and be able to speak and read English. Patients meeting any of the following criteria are excluded: the presence of severe mental, cognitive or physical conditions that would limit the patient’s ability to participate as per treating clinician, lymphoma not in remission (applicable for group 1 only), and patients receiving maintenance treatment.

### Who will take informed consent? {26a}

Treating clinicians will identify potentially eligible patients, obtain their consent to be contacted by the research team, and refer them to the research nurse; the research nurse will contact the patient and provide the patient with full information of the study including its purpose, procedures, expected duration, and the potential benefits, risks, and inconveniences in participation both verbally and in the form of the “Participant Information and Consent Form” (PICF). Prior to being asked to sign the consent form, patients will be given ample opportunity to ask questions and decide whether to participate in the study. All questions about the study will be answered to the satisfaction of the patient. Prior to participation, the written PICF will be signed and personally dated by the patient and the research nurse who conducted the informed consent discussion. The patient will receive a copy of the signed and dated PICF.

### Additional consent provisions for collection and use of participant data and biological specimens {26b}

Not applicable. This trial does not involve collecting additional participant data or biological samples for storage. There are no plans for ancillary studies using data collected in this trial.

## Interventions

### Explanation for the choice of comparators {6b}

With the ever-growing population of cancer survivors, the specialist model of follow-up is insufficient and unsustainable. The shared care model between specialists and GPs focuses on the complex care needs of lymphoma survivors, encompassing the strengths and expertise of multiple providers [10]. It has the potential to contribute to better patient health outcomes as well as reducing the strain on overcrowded hospital-based outpatient services. Effective shared care in the hematology setting relies on establishing and maintaining ongoing communications channels [10]. It is also proposed that transition to primary care occurs via a specialist nurse to ensure key components of survivorship care are addressed and communicated to health care providers [15]. These key considerations have been incorporated in the intervention described below.

### Intervention description {11a}

#### Arm 1

Participants who are randomized to the usual care arm will receive standard follow-up care plus a survivorship booklet on “Living Well After Treatment—A guide for patients and families” published by Leukaemia Foundation [16]. This booklet was designed by our research team in collaboration with expert clinicians and consumers via the Leukaemia Foundation. The current follow-up arrangement is a specialist-led model as determined by the treating hematologist.

#### Arm 2

Gospel I. Participants who are randomized to the GOSPEL I arm will receive a multi-faceted intervention that includes a pre-specified shared care pathway for follow-up. The design of the GOSPEL I is informed by a number of clinical guidelines [i.e., National Comprehensive Cancer Network [17] and European Society of Medical Oncology [18–20]], the Optimal Care Pathway for HL and Diffuse Large B-Cell Lymphoma (DLBCL) [21], the self-efficacy model [22, 23], and the Capabilities for Supporting Prevention and Chronic Condition Self-Management framework [24]. Table 1 outlines the active ingredients of the intervention.

**Table 1:** Active ingredients of GOSPEL I model of care intervention for patients allocated to receive the GOSPEL 1 intervention

Active ingredient	Personnel involved	Activities
**Pharmacy review** Duration: Up to 20 min Mode: Teleconference	Pharmacist and the patient	• Obtain and record medication history including cancer and non-cancer therapy ***Note****: The pharmacy review will be offered to all patients in the intervention arm and will be provided via teleconference or phone call based on the patient’s preference. Medication history completion (Y/N) will be recorded by the RN as a process measure and will therefore not be considered a protocol violation should patients decline this service.*
**SCN-led clinic** Duration: 60 min Mode: Teleconference or face to face	Specialist cancer nurse and the patient	• Treatment summary including follow-up appointment schedule • Co-developing the SCP (including planning for health goals) • Post-treatment education ***Note****: The completed draft SCP will be sent to the GP prior to the case-conference. The research team or SCN will organize the case conference with the GP.*
**GP case conference and optional “booster” case conference** Duration: Maximum 40 min Mode: Teleconference	SCN, GP (± one more healthcare professional).	• The SCN will present the Treatment Summary, and the draft Survivorship Care Plan. • The SCN will negotiate follow-up responsibilities with the GP and will • Answer any questions the GP may have. • Additional education and support to the GP ± practice nurse if applicable regarding physical examination and blood analysis. ***Note****: A copy of the completed and agreed Survivorship Care Plan provided to the GP and the patient and scanned for IeMR. Where a full case conferencing is not possible, all efforts will be made to facilitate a teleconference of a shorter duration (~ 5 min) to deliver all key information on the Survivorship Care Plan. An additional “booster” case conference will be offered to GPs based on their preference should they require further support prior to participating in lymphoma surveillance activities.*
**Standardized shared follow-up care** Mode: Face to face	GP, SCN, patient, hematologist	• The cancer specialist will review the patient history (i.e., fevers, sweats, loss of weight, infections), conduct a physical examination (i.e., lymphadenopathy, hepatosplenomegaly), and order/review blood tests (i.e., FBC, U+Es, LFTs LDH). • GPs who agree to participate in all aspects of follow-up care will review the patient history (i.e., fevers, sweats, loss of weight, infections), conduct a physical examination (i.e., lymphadenopathy, hepatosplenomegaly), and order/review blood tests (i.e., FBC, U+Es, LFTs LDH). • As a safety measure the SCN/RN will ensure that surveillance activity is conducted as per clinical guidelines. Where the GP is unable to complete this activity, the SCN will ensure surveillance activity is transferred to the acute cancer center/specialist. This will be documented as a process measure or future studies in this area. • The GP will also carry out care activities outlined in the SCP. • The GP will follow an escalation pathway which utilizes the SCN as the rapid access point for rapid re-entry to the acute setting and point of contact for the GP for support and resources. ***Note****: The GP will be given the direct telephone number of the SCN responsible for the patient. At any time if the GP becomes concerned about the patient, he/she can ring the SCN for advice or request escalation to acute care for review.*

*Abbreviations*: *SCN* specialist cancer nurse, *SCP* Survivorship Care Plan, *GP* general practitioner, *IeMR* integrated electronic medical records, *FBC* full blood count, *U&E* urea and electrolytes, *LFT* liver function tests, *LDH* lactate dehydrogenase

After enrolment, participants in the intervention arm will receive a consultation with a specialist cancer nurse to provide a treatment summary, the shared follow-up care appointment schedule, and survivorship patient education (including the survivorship booklet on “Living well After Treatment” published by the Leukaemia Foundation) [16] and co-develop a draft Survivorship Care Plan (SCP) with the patient. The SCP will include up to three SMART (Specific, Measurable, Achievable, Realistic and Timely) goals using motivational interviewing and self-efficacy techniques. The treatment summary and draft SCP will be provided to the GP.

Within 4 weeks of the specialist cancer nurse consultation, a case-conference at a mutually agreed time (maximum of 40 min) between the specialist cancer nurse and the patient’s nominated GP will be completed to communicate the treatment summary, negotiate responsibilities of the shared-follow-up care schedule, and negotiate the GP’s role in facilitating the SCP goals. The GP may propose changes or express if they are unable to take part in specific care activities outlined in the SCP. The finalized SCP will be filed in the patient’s medical records and provided to the patient and the GP. As a safety measure, the SCN will ensure that surveillance activities are conducted as per clinical guidelines. Where the GP is unable to complete any of these activities, the SCN will ensure these surveillance activities are transferred to the acute cancer center/specialist. This will be documented as a process measure for future studies in this area.

The GP will also be provided with an escalation pathway which utilizes the SCN as the immediate access point for rapid re-entry to the acute setting and point of contact for the GP for support and resources. The SCN thus plays a key role in providing resources to support the GP ± practice nurse in carrying out all activities outlined in the SCP.

### Criteria for discontinuing or modifying allocated interventions {11b}

The presence of any of the following criteria constitutes cause for the withdrawal of the participant: altered mental capacity resulting in inability to provide continuing informed consent, notification from treating oncologist and or GP that participant is not deemed to have capacity to consent, and recurrence or progressive disease or death.

### Strategies to improve adherence to interventions {11c}

Fidelity of the intervention will be assessed using the framework for behavioral interventions recommended by NIH [25, 26] as outlined in Table 2.

**Table 2:** Framework for behavioral interventions recommended by National Institute of Health

Goal	Strategies
Provider requirements	Intervention nurses must be SCNs. SCNs work in dedicated cancer services and are primarily responsible for care of people at a specific phase or across all phases of the cancer journey or work in a broader context but provide a specialist resource in cancer control to a range of generalist providers (for example, a cancer nurse coordinator). SCNs meet the minimum standard required for specialist practice in cancer nursing as set out in the competency standards from Cancer Australia [27]. Therefore, the nurse delivering the GOSPEL I intervention requires the critical thinking, coordination, and collaboration competencies defined in the SCN competency standards.
Training providers	Training will be provided to the SCNs to ensure standardization of intervention delivery. Provision of a study manual to all SCNs which includes: • Generic study related information: standard operating procedures, study overview, reporting/documentation guidelines, communication flowchart, rationale for the study treatment, completion of survivorship care plan, self-management goal setting, and health coaching (including motivational interviewing) resources • Interventionist specific information: job description, intervention protocol, quality assurance, and monitoring processes Completion of the eviQ Cancer Survivorship Introductory Course (~ 4.5 h over 6 modules). This course was developed by the Australian Cancer Survivorship Centre in collaboration with Cancer Australia, Queensland University of Technology, and the University of Sydney and is available online free of charge. Completion of face-to-face training from the research team which includes self-management support in cancer care, motivational interviewing techniques, and setting SMART goals. Intervention-specific procedures required for this trial. Education and resources regarding MBS item numbers that facilitate the proposed Model of Care. Resources to support GPs in performing physical examination and blood analysis.
Delivery of intervention	Intervention procedures are monitored through completion of intervention component checklists to ensure that the intervention is delivered as intended. Intervention checklists are completing during the SCN-led clinic and GP case conferences to track protocol deviations. All nurse-led clinics will be recorded and checked by another member of the research team against the clinic checklist to allow protocol deviation tracking across interventionists and conditions. Minimizing contamination between conditions by training SCNs to address participant questions about randomization and their assigned condition using non-biased explanations. SCNs will be supported during a weekly 15–30-min meeting for the first 3 months of the trial between the SCN and CIA or project manager. In addition to ongoing troubleshooting and support, intervention fidelity will be closely monitored and will be discussed during this meeting.
Receipt of intervention	The SCP serves as a resource for a participant to understand and refer to whenever they are unsure of follow-up schedule and collaborative goal setting.
Enactment of treatment skills	Enactment of treatment skills includes processes to monitor and improve participant ability to perform treatment-related behavioral skills and cognitive strategies in relevant real-life settings as intended. This goal will be achieved by: - Ensuring participants are aware of the follow-up schedules and responsibilities of all health professionals - Ensuring participants will have a copy of the completed self-management care plan including all care responsibilities and goals set for the individual

### Relevant concomitant care permitted or prohibited during the trial {11d}

No concomitant care or intervention is prohibited during the trial.

### Provisions for post-trial care {30}

There is no specified ancillary or post-trial care for participants in this trial. However, it is expected that the SCP generated will have the value of informing longer-term updates of the SCP and future survivorship care.

### Outcomes {12}

The feasibility outcomes are recruitment and acceptability of the intervention based on completion rates and semi-structured interviews. Participants or healthcare providers who opt into the 12-month semi-structured interview will be interviewed either face-to-face, by telephone, or through videoconferencing as per interviewee preference.

A range of patient-reported and process outcome measures will also be collected. Health-related quality of life as measured by the Functional Assessment of Cancer Therapy—Lymphoma (FACT-Lym) will be collected at baseline and 6 and 12 months post enrolment [28]. This valid and reliable tool captures key domains of health-related quality of life relevant to lymphoma and key symptoms that are relevant to the study population and sensitive to the GOSPEL I intervention. Additional outcomes include a range of patient-reported and process outcomes related to implementation as shown in Table 3.

**Table 3:** Schedule for data collection during the GOSPEL 1 trial

	Study period					
	Enrolment	Allocation	Post-allocation			Close-out
Timepoint	Pre-baseline	Baseline	Baseline	6 months*	12 months*	18 months
**Enrolment**						
Eligibility screen	X					
Informed consent	X					
Allocation		X				
**Interventions**						
GOSPEL 1						
Usual care						
**Outcomes**						
Recruitment (feasibility)					**X**	
Barriers and facilitators of care: optional interviews (feasibility)					**X**	
Satisfaction of care					**X**	
Medications history			**X**			
HRQoL (FACT-Lym)			**X**	**X**	**X**	
Patient experience of care			**X**	**X**	**X**	
Symptom distress			**X**	**X**	**X**	
Comorbidity burden			**X**		**X**	
Dietary intake			**X**	**X**	**X**	
Physical activity			**X**	**X**	**X**	
Financial distress			**X**	**X**	**X**	
Employment interference			**X**	**X**	**X**	
**Participant characteristics**						
Demographics			**X**			
Clinical characteristics			**X**			
**Process Outcomes**						
Intervention fidelity: completion of checklists						**X**
Clinical encounters at cancer center						**X**
Cost analysis: resources to conduct the intervention						**X**
Safety indicators: clinical encounters, unscheduled clinic visits and rapid referrals back to acute care						**X**

*Post-baseline

### Participant timeline {13}

### Sample size {14}

In this pilot study, we will recruit 30 patients per arm in order to provide initial insights into the intervention feasibility and protocol as well as preliminary effect size estimates. The aim of this study is not hypothesis testing; the power level is therefore not a valid consideration for sample size [29, 30]. The sample size for this study (*n* = 60) falls within the range of sample size recommendations for pilot studies of this nature [29, 30].

### Recruitment {15}

Potentially eligible patients will be identified by clinicians and/or the research nurse through attendance at multidisciplinary team meetings and utilizing an existing clinical database. Potentially eligible patients will be identified; the treating clinician will ask the potential participant whether they agree to being contacted by the research nurse and will advise the research team accordingly. A patient brochure has been produced for clinicians to use when first discussing the study with potential participants. Only patients who have agreed to be contacted will be approached by the research nurse. Participants are given as much time as possible to consider their participation and are encouraged to take the information away and discuss joining the trial with family, friends, and their GP if they so wish to. Participants are also encouraged to ask the research nurses, their treating doctors, or nursing staff any questions in relation to their participation.

### Assignment of interventions: allocation

#### Sequence generation {16a}

**Fig. 1:**
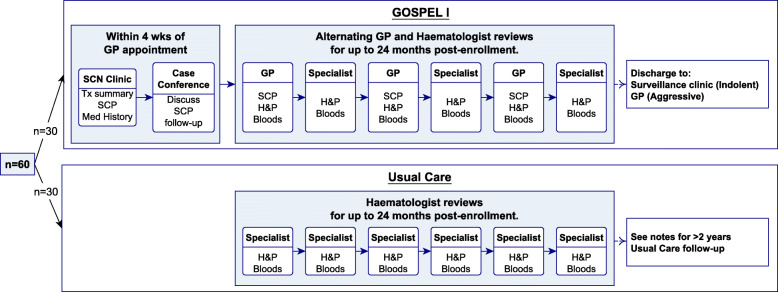
Comparison of usual care with standardized frequency and timing of follow-up for GOSPEL I components. Med history will be informed by pharmacy review prior to the SCN Clinic. Three-monthly follow-up schedule: flexibility 3 weeks ± the scheduled GP and specialist appointment permitted. Six-monthly follow-up schedule: flexibility 8 weeks ± the scheduled GP and specialist appointment permitted. > 2 years follow-up for usual care: indolent lymphoma, ongoing 6-monthly specialist review; aggressive lymphoma, 6-monthly specialist review (years 2–3), annual specialist review (years 3–5), discharge to GP after 5 years. SNC, Specialist Cancer Nurse; Tx, treatment; SCP, Survivorship Care Plan; Med, mediation; GP, general practitioner; H&P, history and physical examination. “History” includes review of patient experience of fevers, sweats, loss of weight, infections; “Physical Examination” includes check for lymphadenopathy, splenomegaly ± hepatomegaly; “Bloods” includes order and review of full blood count, urea and electrolytes, liver function tests, and lactate dehydrogenase. If the patient’s hematologist notes that additional clinic visits are clinically indicated, then they can schedule additional appointments

Permuted block randomization will be conducted to assign participants to the control or intervention arms (Fig. 1). To ensure equal distribution of patients with different follow-up schedules, patients will be stratified by diagnosis (NHL vs HL) and follow-up pathway (group one: post-treatment vs group two: surveillance clinic).

#### Concealment mechanism {16b}

Allocation sequence is implemented using sequentially numbered opaque, sealed envelopes. Envelopes are only accessed by the research nurse to randomize the patient once recruitment and baseline data has been collected.

#### Implementation {16c}

Allocation sequence is generated by a researcher not involved in recruitment or data collection. Patients are enrolled by a research nurse who collects baseline data prior to randomization. Enrolling nurses assign participants to the intervention after baseline data collection.

### Assignment of interventions: blinding

#### Who will be blinded {17a}

After assignment to the intervention, only outcome assessors and data analysts are blinded to group allocation. Where participants opt to complete their data collection by phone, they are advised not to reveal their group allocation to the outcome assessor. Due to the nature of the intervention, no participants or treating clinicians are blinded.

#### Procedure for unblinding if needed {17b}

No unblinding procedures required as only outcome assessors and data analysts are blinded.

### Data collection and management

#### Plans for assessment and collection of outcomes {18a}

Patient-reported outcomes are self-administered using online surveys or administered in person or via telephone with an outcome assessor trained in the administration of the study instruments.

A range of feasibility, patient-reported, and process outcomes related to implementation will be collected as shown in Tables 3 and 4.

**Table 4:** Study outcome definitions

Outcome domain	Specific measurement	Metric and method of aggregation	Time point of interest
Health-related quality of life	The Functional Assessment of Cancer Therapy—Lymphoma (FACT-Lym) [28]	Effect of time on mean change score between groups	Baseline, 6 months, 12 months
Patient experience of care	Patient Assessment of Care for Chronic Conditions (PACIC)	Effect of time on mean change score between groups	Baseline, 6 months, 12 months
Symptom distress	Memorial Symptom Assessment Scale (MSAS) [31]	Effect of time on mean change score between groups	Baseline, 6 months, 12 months
Comorbidity burden	The Charleston Comorbidity Index (CCI) supplemented with items from Self-Administered Comorbidity Questionnaire (SCQ) [32]	Difference between mean change scores from baseline	12 months
Total leisure-time physical activity	Active Australia Survey [33]	Effect of time on mean change score between groups	Baseline, 6 months, 12 months
Usual vegetable intake and usual fruit intake	Two short dietary questions from the National Nutrition Survey [34]	Effect of time on mean change score between groups	Baseline, 6 months, 12 months
Financial distress	0–10 numerical analogue scale (where 10 is “a great deal” and 0 is “none”) [35]	Effect of time on mean change score between groups	Baseline, 6 months, 12 months
Employment interference	0–10 numerical analogue scale (where 10 is “a great deal” and 0 is “none”) [36]	Effect of time on mean change score between groups	Baseline, 6 months, 12 months
Satisfaction of care	0–10 numerical analogue scale (where 10 is “most satisfied” and 0 is “least satisfied”)	Difference in mean score between groups	12 months

Barriers and facilitators of care as measured by voluntary participation in semi structured interviews 12 months post enrolment. All intervention arm patients, lymphoma cancer nurses, GPs, and other nurses, and hospital- and community-based rehabilitation providers will be invited to participate in a one off semi-structured interview to discuss factors that facilitated or hindered the implementation of the GOSPEL I intervention.

Process outcomes, including completion of intervention components, as measured by completion of intervention materials such as clinic checklists, audio recordings of SCN-led clinics, and research nurse (RN) records in database will be checked for completion (Y/N) and duration (min) of SCN-led clinics.

Cost analysis will be conducted using hospital casemix data describing occasions of service including duration and indication and RN records in database. All resources required to conduct the intervention (e.g., staff, training, materials, communications, office space, utilities) will be monitored.

Safety indicators will be measured via hospital records to query the number of hospital clinical encounters, unscheduled lymphoma clinic visits, and rapid referrals back to acute care.

#### Plans to promote participant retention and complete follow-up {18b}

Participants who deviate from the protocol will not be withdrawn from the trial. Participants who withdraw from the trial nominate the degree to which they withdraw (i.e., whether they withdraw from active data collection ± passive data collection such as hospital records). To avoid losing participants to follow-up, contact information of a friend or family member will be requested as a back-up contact in case of difficulty in contacting the participant. Provision of a contact is not mandatory and will not result in exclusion from the trial.

#### Data management {19}

All participant characteristic and outcome data are entered directly into REDCap (Research Electronic Data CAPture—Vanderbilt University, hosted at Queensland University of Technology) by the research nurse and the participants through self-administered online survey. To ensure data quality, the database is designed with branching logic, data validation, and range checks for data values, where possible.

All source data, clinical records, and laboratory data relating to the study will be archived at the clinical site as appropriate for 15 years after the completion of the study. All data will be available for retrospective review or audit. No study document will be destroyed without prior written agreement between the responsible organization and the investigator. If the investigator wishes to assign the study records to another party or move them to another location, he/she must notify the responsible organization in writing of the new responsible person and/or the new location.

#### Confidentiality {27}

Data on potential participants is recorded, including reasons for ineligibility or refusal to participate. Participants are only identified by a unique participant study number on the case report forms and other study documents. Other study-related documents (e.g., signed consent form, participant log) are kept in strict confidence by the investigator.

Participants are informed that data is held on file by the responsible organizations and that these data may be viewed by staff including the study project manager and by external auditors on behalf of the responsible organizations and appropriate regulatory authorities (to include reviewing Human Research Ethics Committee (HREC) and the Research Governance Officers). Participant data in publications and conference presentation reports will only be presented in aggregated form. All participant data will be held in strict confidence.

#### Plans for collection, laboratory evaluation, and storage of biological specimens for genetic or molecular analysis in this trial/future use {33}

Not applicable. There is no collection of biological specimens in the current trial.

## Statistical methods

### Statistical methods for primary and secondary outcomes {20a}

Descriptive statistics will be used to report on feasibility and process-related elements (e.g., recruitment, intervention, retention rates) as well as clinical and resource outcomes. Preliminary effect size estimates for patient and resource use outcomes will be calculated following intention-to-treat principles using linear mixed models. Models will include group, time, and their interaction and be adjusted by diagnosis and age. Balance of demographic variables between usual care and intervention group will be investigated using chi-square and *t* test and will be included in the model if found to be both significantly associated with the outcome and confounding the intervention. Assumptions of all models (normality, linearity, homoscedasticity) will be examined using the residuals of the model and will be described using mean, median, skewness, kurtosis, and plots such as histograms and QQ-plots. If assumptions are violated, models will be either bootstrapped or log transformation as appropriate.

### Interim analyses {21b}

Not applicable. No interim analysis is planned.

### Methods for additional analyses (e.g., subgroup analyses) {20b}

Patients allocated to the GOSPEL I arm will be invited to participate in an interview at the 12-month time point. Guiding questions and analysis of the interviews will be guided by the Consolidated Framework for Implementation Research (*CFIR*)*.* Based on our previous qualitative work, we expect that the number of interviews will be approximately 24 (GPs, patients, hematologists). All interviews will be recorded and transcribed verbatim for analysis.

### Methods in analysis to handle protocol non-adherence and any statistical methods to handle missing data {20c}

Any discrepancies and missing data will be alerted and resolved with the relevant research team member(s) as soon as practical. All electronic CRFs will be maintained on the system with details of any changes logged accordingly. Preliminary effect size estimates for patient and resource use outcomes will be calculated following intention-to-treat principles using linear mixed models. Patterns of missing data will be examined using chi-square and *t* tests. Missing data for the outcomes will be accounted for by using mixed models allowing the use each available case by computing maximum likelihood estimates.

### Plans to give access to the full protocol, participant level-data and statistical code {31c}

Not applicable. There are no plans for granting public access of the full protocol, participant level dataset or statistical code.

## Oversight and monitoring

### Composition of the coordinating center and trial steering committee {5d}

The chief investigators are the trial steering committee that will provide all governance to the conduct of the study. There are no other trial committees.

### Composition of the data monitoring committee, its role and reporting structure {21a}

Not applicable. There is no data monitoring committee established for this pilot trial.

### Adverse event reporting and harms {22}

An adverse event (AE) is any event, side effect, or other untoward medical occurrence that occurs in conjunction with the use of the study intervention in humans, whether or not considered to have a causal relationship to the interventions. An AE can, therefore, be any unfavorable and unintended sign (that could include a clinically significant abnormal laboratory finding), symptom, or disease temporally associated with the use of the study intervention, whether or not considered related to the intervention. Conditions recognized as being excluded from AE reporting are as follows: any event, side effect, or other medical occurrence that is anticipated because of the normal course of treatment (standard care). There are no known side effects/adverse events associated with the proposed model of care intervention [37]. Due to the nature of this intervention, there will be no reporting of AE.

### Frequency and plans for auditing trial conduct {23}

There are no plans for auditing trial conduct beyond the independent research governance requirements and annual reporting to the HREC.

### Plans for communicating important protocol amendments to relevant parties (e.g., trial participants, ethical committees) {25}

All agreed protocol amendments are clearly recorded on a protocol amendment form and are signed and dated by the original protocol approving signatories. All protocol amendments will be submitted to the institutional HREC for approval before implementation. The only exception will be when the amendment is necessary to eliminate an immediate hazard to the trial participants. In this case, the necessary action will be taken first, with the relevant protocol amendment following shortly thereafter. Once HREC approval has been granted, investigators and the ANZCTR will be updated.

### Dissemination plans {31a}

It is intended that the findings from this trial will be disseminated at academic and professional conferences and via a manuscript submission to a peer-reviewed journal. Participants will be identified in such reports only in aggregate or by study identification number, gender, and age. There are no publication restrictions. Authorship will be discussed between researchers prior to study commencement (or as soon as possible thereafter) and reviewed whenever there are changes in participation. All conflicts arising through disputes about authorship will be reviewed by the HREC.

## Discussion

Despite the strong case for shared, follow-up care model for lymphoma survivors involving cancer specialists and GPs, barriers to shared care mean that it is not routinely implemented across Australia. These include the need for coordination across multiple providers, the need for improved patient and provider knowledge about the benefits of shared care and how to implement it, insufficient or delayed communication between cancer specialists and GPs, and lack of awareness of available support such as funding models, tools and resources [11, 12]. Facilitated by the specialist cancer nurse the current study aims to help stakeholders (patient, hematologist and GP’s) realize the benefits of shared care (patient and GPs), facilitating effective and timely care coordination, and acting as the conduit between the specialist cancer multidisciplinary team and the GPs.

Practical issues for this trial include estimating the time required to coordinate the trial across multiple providers including engaging GPs and fidelity with the intervention components. The proposed study will provide important information on the feasibility of a definitive phase 3 trial to advance the evidence to support an integrated model of care that will ultimately optimize outcomes for patients with lymphoma. The information collected through the trial, qualitative interviews, and economic evaluations are crucial in guiding the development of such a trial.

### Trial status

The protocol published here is version 1.5 dated 3 August 2020. The trial commenced recruitment as of 24 July 2020. Estimated end date of recruitment is 24 July 2021.

#### Trial registration

Australia and New Zealand Clinical Trials Registry, registration number: ACTRN12620000594921, date registered: 22 May 2020, registration link: https://www.anzctr.org.au/ACTRN12620000594921.aspx

## Supplementary Information

**Additional file 1.**

## Abbreviations

AE: Adverse event
CCI: Charleston Comorbidity Index
CRF: Case report form
ECOG: Eastern Cooperative Oncology Group
FACT-Lym: Functional Assessment of Cancer Therapy—Lymphoma
HREC: Human Research Ethics Committee
HRQoL: Health-related quality of life
HL: Hodgkin’s lymphoma
ieMR: Integrated electronic medical record
IPAQ: International Physical Activity Questionnaire
MSAS: Memorial Symptom Assessment Scale
NHL: Non-Hodgkin’s lymphoma
PACIC: Patient Assessment of Care for Chronic Conditions
PICF: Patient Information and Consent Form
QUT: Queensland University of Technology
RCT: Randomized controlled trial
REDCap: Research Electronic Data Capture
RN: Research nurse
SCP: Survivorship Care Plan
SCQ: Self-Administered Comorbidity Questionnaire
T1: Timepoint 1 (baseline)
T2: Timepoint 2 (6 months)
T3: Timepoint 3 (12 months)
T4: Timepoint 4 (24 months)
Y/N: Yes/no
