# Supplementary material for: Partnering with general practitioners to optimize survivorship for patients with lymphoma: a phase II randomized controlled trial (the GOSPEL I trial)
Source: Trials. 2021 Jan 6;22:12. doi: 10.1186/s13063-020-04945-4 (PMC7787240; doi:10.1186/s13063-020-04945-4)
Supplement: Supplementary file 1 — Additional file 1. [file 13063_2020_4945_MOESM1_ESM.pdf]

Participant Information and Consent Form

Title	Partnering with General practitioners to Optimise Survivorship for PatiEnts with Lymphoma: A Phase II randomised controlled trial (The GOSPEL Trial)	
Short title	The GOSPEL Trial	
Protocol number	HREC/2020/QMS/61872	
Responsible Organisation	Princess Alexandra Hospital, Metro South Health	
Investigators	Professor Raymond Chan, Dr Sally Mapp, Professor Jane Turner, Associate Professor Louisa Gordon, Ms Lee Jones, Ms Stephanie Buhagiar, Ms Camilla Simonsen, Ms Laisa Teleni, Associate Professor Joel Rhee, Ms Marissa Ryan, Dr Christine Carrington	
Research team contact	Laisa Teleni<br>Camilla Simonsen<br>Courtney Rawson	Telephone: 0466 628 401<br>Email: cancersurvivorship@qut.edu.au<br>Email: Courtney.rawson@health.qld.gov.au

1. Would you like to take part in this clinical trial?

We would like to invite you to take part in our clinical trial. This is because **you have lymphoma and attend the surveillance clinics OR you have completed treatment in the last three months at the Princess Alexandra Hospital**. This document tells you about the trial and describes what will happen if you decide to take part. If there is anything you don't understand or want to know more about, please ask us. We will be happy to provide more information. If you do not know what to ask, there are some questions to consider in the *Clinical trial participant information and consent form: Part A – General information.* You might also want to talk to a relative, a friend or your doctor before you make up your mind. If you decide to go ahead, we will ask you to sign the consent form (the signature pages of this document). We will give you a copy of the complete signed document to keep.

2. Why are we doing this research?

In this study, we are trying to test whether a **type of follow-up care led by a nurse (which involves your Haematologist, a specialist nurse, an oncology pharmacist and your general practitioner (GP))**, is feasible and acceptable to you and whether the follow-up care has any benefits to your quality of life or healthcare costs. The current models of care often require patients to frequently attend the cancer centre. The follow-up appointment schedules and the involvement of each healthcare practitioner (including the GP) can vary from patient-to-patient, depending on the facility where they are being seen and as decided by each healthcare practitioner. Our research team proposes that patients' health and experience of care would benefit from a more consistent model that involves all cancer specialists as well as the GP and uses video calls. We propose that a standardised, coordinated model of follow-up care involving your cancer specialists and GP would be beneficial for your health and experience of care.

3. What does participation in this research involve?

If you agree to participate in this study, you agree to be responsible for complying with the conditions in this document. If you cannot, or do not wish to accept this responsibility, then we cannot accept you as a participant in the study. You can advise us at any time if you wish to withdraw from the study.

If you decide you want to take part in the research project, you will be asked to sign the Consent Form. By signing it you are telling us that you:

- You understand what participation involves
- You give your permission for the research team to notify your treating Haematologist and GP of your participation in, or withdrawal from, the study and for the research team to notify them of anything which affects your medical treatment or care
- You give your permission for the research team, health care providers, Human Research Ethics Committee and Research Governance Officers to access your medical records held by Australian public and private hospitals and health services for the duration of this study
- If you withdraw from the study, any information collected up to the time you withdraw will be retained

3.1. What do I need to do?

If you agree to participate, a member of the research team will provide you with detailed information about the study within the last three months of your treatment (or any time if you attend the lymphoma clinic for surveillance). You will be randomly allocated to one of the two treatment groups: *GOSPEL I* or Usual Care. The process of random allocation will allow this research project to effectively test the feasibility, acceptability and potential effects of the different follow-up models of care.

This research project has been designed to make sure the researchers interpret the results in a fair and appropriate way and avoids study doctors or participants jumping to conclusions. There are no additional costs associated with participating in this study, nor will you be paid. If you decide to participate in this study, the research team will inform your GP.

If you are randomised to receive *GOSPEL I* you will receive a pharmacist appointment, Specialist nurse appointment followed by alternating GP and Haematologist appointments.

<u>Oncology Pharmacist appointment (up to 20 min)</u>

An oncology pharmacist will go through your medications with you to make sure your GP is given up to date information about the medications you are currently taking. This appointment can be by telephone or videoconferencing, depending on your preference.

Specialist nurse appointment (40-60 min)

You will be secheduled to see a specialist cancer nurse soon after the pharmacy appointment. The timing of this appointment will depend on whether you have received treatment or are under ongoing surveillance for lymphoma. This appointment can be face-to-face, by telephone or by videoconferencing, depending on your preference. All of these appointments will be recorded.

During this appointment, your nurse will provide you with your treatment summary, follow-up appointment schedule, and education about symptom management, healthy lifestyle and the "Living Well After Treatment – A guide for patients and families" booklet published by Leukaemia Foundation. The nurse will also work with you to develop a Survivorship Care Plan including your goals and a list of the responsibilities of all healthcare providers involved (including your hospital cancer care team and your GP).

Before your first appointment with your GP, your specialist nurse will communicate all care you received in the cancer centre including the treatment summary and Survivorship Care Plan with your GP, and seek his/her input.

Alternating GP & Haematologist appointments

You will be provided with a schedule of appointments for your GP and Haematologists for up to 2 years. The duration of these appointments will be up to you and your GP/Haematologist. You are not required to memorise all appointments as these will be listed in the Survivorship Care Plan, and this plan will be provided to you and your healthcare professionals. During these appointments the Haematologist or GP will ask you about your symptoms, conduct a physical exam of your lymph nodes and spleen and order and review your blood tests. During the GP appointments, your GP will also discuss your Survivorship Care Plan. Should your GP decline certain activities, your specialist nurse will ensure these are done at the cancer centre/by the specialist. **If you are randomised to receive 'Usual Care'** you will be cared for by your Haematologist, nurses and other healthcare professionals as usual. There will be no change to your care except, we will provide you with an additional information booklet on "Living Well After Treatment – A guide for patients and families" published by Leukaemia Foundation.

3.2. Complete Questionnaires

Whether you receive *GOSPEL I* or Usual Care, a member of the research team will contact you at three points during the study period to ask you to complete questionnaires so that we can find out about your quality of life, lifestyle and experiences of care before you start the study, at 6 months and 12 months after you commenced on the study. The questionnaires will take about 20-30 minutes to complete each time. The research team will send reminders to you via phone call, text or email, as required. It should be noted that the questions will not be reviewed by a health professional and you will need to contact an appropriate practitioner (i.e. your specialist nurse) if you

have concerns about your health. Your specialist nurse will provide you with this number at the completion of your cancer treatment.

3.3. Complete Interview (30 minutes, optional)

A member of the research team will contact you 12 months after you commenced on the study and invite you to participate in a one off, individual interview to find out about your experience of participating in the study. This interview is completely voluntary and will be recorded to allow the research team to reflect and analyse the interview data later. The interview should take no longer than about 30 minutes.

4. Do I have to take part?

Participation in any research project is voluntary. If you do not wish to take part, you do not have to. You will receive the best possible care whether or not you take part. If you do decide to take part, you will be given this Participant Information and Consent Form to sign and you will be given a copy to keep. If you decide to take part and later change your mind, you are free to withdraw from the study at any stage. Your decision whether to take part or not to take part, or to take part and then withdraw, will not affect your routine treatment, your relationship with those treating you or your relationship with the Princess Alexandra Hospital.

5. What are the alternatives to participation?

You do not have to take part in this research project to receive treatment at this hospital. Other options are available; these include usual follow-up care, like participants in the control arm. The research team will discuss these options with you before you decide whether or not to take part in this study. You can also discuss the options with your GP.

6. What are the possible benefits of taking part?

We cannot guarantee or promise that you will receive any personal benefits from this research. It is possible that you may experience an improvement in your health and experience of care from the holistic, structured, frequent review by your GP during the study. By taking part in this study, you may be helping other people in the future by helping us find the most effective and satisfactory way of providing follow-up care.

7. What are the possible risks of taking part?

There are minor risks associated with your participation in this project as outlined below:

It is possible that your GP may not be as confident in performing reviews of people with lymphoma. As a safety measure, your specialist nurse will discuss all aspects of your ongoing care with your GP. Should your GP decline certain activities, your specialist nurse will ensure these are done at the cancer centre/by the specialist. This risk is minimised by ensuring that you continue to attend your

scheduled appointments with your Haematologist. In additon, if your Haematologist thinks that they need to see you more frequently, then they can schedule additional appointments with you. The specialist cancer nurse will also provide education and support to the GP and act as a point of rapid referral. This means if your GP identifies any concerns, he/she will be able to rapidly refer you back to your Haematologist by contacting your specialist cancer nurse. There is a very small possibility that you might experience distress during the questionnaires or if you choose to participate in our interview at the end of the study. Our questionnaires ask about your quality of life, symptoms, disease, and our questionnaires and interviews ask about your experience of care. If you become upset or distressed as a result of your participation in the study, the research team will be able to arrange for counselling or other appropriate support. Any counselling or support will be provided by qualified staff who are not members of the research project team. This counselling will be provided free of charge. Alternatively, Lifeline provides access to online, phone or face-to-face support, call 13 11 14 for 24-hour telephone crisis support.

8. What if new information arises during this research project?

Sometimes during the course of a study, new information becomes available about the treatment that is being studied. If this happens, the research team will tell you about it and discuss with you whether you want to continue in the study. If you decide to withdraw, the research team will make arrangements for your regular health care to continue. If you decide to continue in the study, you will be asked to sign an updated consent form.

Also, on receiving new information, the research team might consider it to be in your best interests to withdraw you from the study. If this happens, the research team will explain the reasons and arrange for your regular health care to continue.

9. What if I withdraw from this research project?

If you decide to withdraw from the study, please notify a member of the research team before you withdraw. This notice will allow that person or the research supervisor to discuss any health risks or special requirements linked to withdrawing. If you do withdraw your consent during the research project, the research team will not collect additional personal information from you.

10. What happens when the research project ends?

We will not contact you again after the 2-year follow-up period for this study. At 2 years, you will either be discharged to the care of your GP or will continue to attend lymphoma clinics for surveillance as is usual practice, depending on the type of lymphoma you have.

11. What will happen to information about me?

By signing the consent form, you consent to the research team collecting and using personal information about you for the study. Any information obtained in connection with this study that can

identify you will remain confidential. All the collected data will be coded. No personal information about you, such as your name and address will be used outside of this study. Your information will be identified with a code number only. All your collected information will be kept for at least 15 years after the end of the study. After the 15 years your identifying information will be permanently deleted from the computer system and any hard copies will be destroyed making your data deidentified (i.e. unable to be linked back to you).

Your information will only be used for the purpose of this study and it will only be disclosed with your permission, except as required by law.

Your health records and any information obtained during the study are subject to inspection (for the purpose of verifying the procedures and the data) by the relevant authorities and authorised representatives of the Sponsor, Professor Raymond Chan, the institution relevant to this Participant Information Sheet, Princess Alexandra Hospital, or as required by law. By signing the Consent Form, you authorise release of, or access to, this confidential information to the relevant study personnel and regulatory authorities as noted above.

It is anticipated that the results of this study will be published and/or presented in a variety of forums. In any publication and/or presentation, information will be provided in such a way that you cannot be identified, except with your permission. Published results will be presented in aggregate.

Information about your participation in this research project will be recorded in your health records. In accordance with relevant Australian and Queensland privacy and other relevant laws, you have the right to request access to your information collected by the research team. You also have the right to request that any information with which you disagree be corrected. Please contact the study team member named at the end of this document if you would like to access your information.

Any information obtained for the purpose of this study that can identify you will be treated as confidential and securely stored. It will be disclosed only with your permission, or as required by law.

12. Who is organising and funding the research?

This study is being conducted by Professor Raymond Chan. No member of the research team will receive a personal financial benefit from your involvement in this research project (other than their ordinary wages).

13. Who has reviewed the research project?

All research in Australia involving humans is reviewed by an independent group of people called a Human Research Ethics Committee (HREC). The ethical aspects of this study have been approved by the HREC of Metro South HREC. This study will be carried out according to the National Statement

on Ethical Conduct in Human Research (2007). This statement has been developed to protect the interests of people who agree to participate in human research studies.

14. What if I have a question or need to make a complaint?

We have included several contacts for you below. The person you may need to contact will depend on the nature of your query.

If you want any further information concerning this study or if you have any medical problems which may be related to your involvement in the project (for example, any side effects), you can contact the research team at any time:

- Camilla Simonsen / Courtney Rawson (Research Nurses): Telephone: 0466 628 401
- Laisa Teleni (Research Coordinator): Telephone: 0466 628 401

The Princess Alexandra Hospital is committed to research integrity and the ethical conduct of research projects. However, if you wish to discuss the study or with someone not directly involved, particularly in relation to matters concerning policies, information or complaints about the conduct of the study or your rights as a participant, you may contact:

Reviewing HREC	Metro South Health Human Research Ethics Committee (EC00167)
Contact Person	HREC coordinator
Telephone	+61 7 3443 8049
Email	MSH-Ethics@health.qld.gov.au
HREC Reference Number	HREC/2020/QMS/61872

Governance Office	Metro South Health Research Governance Office
Contact Person	Research Governance Officer
Telephone	+61 7 3443 8050
Email	MSH-RGO@health.qld.gov.au

Consent form

Title	Partnering with General practitioners to Optimise Survivorship for PatiEnts with Lymphoma: A Phase II randomised controlled trial (The GOSPEL Trial)	
Short title	The GOSPEL Trial	
Protocol number	1.2	
Responsible Organisation	Princess Alexandra Hospital, Metro South Health	
Investigators	Professor Raymond Chan, Dr Sally Mapp, Prof Jane Turner, Associate Professor Louisa Gordon, Ms Lee Jones, Ms Stephanie Buhagiar, Ms Camilla Simonsen, Ms Laisa Teleni, Associate Professor Joel Rhee, Ms Marissa Ryan, Dr Christine Carrington	
Research team contact	Laisa Teleni<br>Camilla Simonsen<br>Courtney Rawson	Telephone: 0466 628 401<br>Email: cancersurvivorship@qut.edu.au<br>Email: Courtney.rawson@health.qld.gov.au

Declaration by participant

- I have read, or have had read to me, and understand the participant information and consent form.
- I have had an opportunity to ask questions and am satisfied with the answers I have received.
- I freely agree to participate in this trial as described and understand that I am free to withdraw at any time during the study without affecting my future health care.
- I understand the purposes, procedures and risks of the research described in the trial.
- I understand that during the course of this research records held by PAH may be accessed by my health care providers, Human Research Ethics Committee, the research team, and Research Governance Officers to determine my eligibility for participation in this trial and for the purposes of conducting and monitoring the trial and verifying results.
- I give permission for my doctors, other health professionals, hospitals, laboratories or ambulances to release information held in my medical and health records to PAH concerning my disease and treatment for the purposes of this trial. I understand that such information will remain confidential.
- I understand that my information collected as part of this study may be used for secondary analysis for another research purpose. When this occurs, the researchers will seek appropriate ethics clearance and ensure the maintenance of my privacy.
- I consent to my treating doctor/s being notified of my participation in this study and any clinically relevant information noted by the trial nurse in the conduct of the trial.
- I understand if I have any queries I can contact the research team on 0466 628 401 or via email at cancersurvivorship@qut.edu.au / Courtney.rawson@health.qld.gov.au
- I understand that I will be given a signed copy of this document to keep. We may like to ask you to participate in a future related study, or to obtain additional information or clarification related to your participation in this study. Please indicate below whether you are willing to be contacted about any future research studies.

(Continued over)

(Continued from previous page)

Future Studies

☐ Yes, I agree to be contacted about future research studies.

☐ No, I do not want to be contacted about future research studies.

Study Results

☐ Yes, I would like a copy of the study results

☐ No, I do not want a copy of the study results

**Signature**	______________________________	**Date**	______________
**Name of participant**	______________________________<br>(please print)	**Time**	______________

Note: All parties signing the consent section must date and time their own signature.

Withdrawal of Consent form

Title	Partnering with General practitioners to Optimise Survivorship for PatiEnts with Lymphoma: A Phase II randomised controlled trial (The GOSPEL Trial)	
Short title	The GOSPEL Trial	
Protocol number	1.5	
Responsible Organisation	Princess Alexandra Hospital, Metro South Health	
Investigators	Professor Raymond Chan, Dr Sally Mapp, Prof Jane Turner, Associate Professor Louisa Gordon, Ms Lee Jones, Ms Stephanie Buhagiar, Ms Camilla Simonsen, Ms Laisa Teleni, Associate Professor Joel Rhee, Ms Marissa Ryan, Dr Christine Carrington	
Research team contact	Laisa Teleni<br>Camilla Simonsen<br>Courtney Rawson	Telephone: 0466 628 401<br>Email: cancersurvivorship@qut.edu.au<br>Email: Courtney.rawson@health.qld.gov.au

Declaration by participant

I wish to withdraw my consent to participate in the above research project and understand that such withdrawal will not jeopardise any treatment or my relationship with the research team or my treating health professionals.

Name of Participant ____

Signature ____

Date ________________ **Time** ________________

This Withdrawal of Participation should be forwarded to the research team contact listed above.
